# Pre-pubertal males practising Taekwondo exhibit favourable postural and neuromuscular performance

**DOI:** 10.1186/s13102-016-0040-2

**Published:** 2016-06-04

**Authors:** Mohamed Chedly Jlid, Nicola Maffulli, Nisar Souissi, Mohamed Souheil Chelly, Thierry Paillard

**Affiliations:** Research Unit of Sport Performance and Health, Higher Institute of Sport and Physical Education of Ksar Said, Tunis, Tunisia; Department of Musculoskeletal Disorders, Faculty of Medicine and Surgery, University of Salerno, 84081 Baronissi, Salerno, Italy; Centre for Sports and Exercise Medicine, Mile End Hospital, Barts and The London School of Medicine and Dentistry, London, UK; Unité de Recherche Evaluation, Sport, Santé, Centre National de Médecine et Science en Sport, Tunis, Tunisie; Laboratoire Activité Physique, Performance et Santé (EA 4445), Université de Pau et des Pays de l’Adour, Département STAPS, ZA Bastillac Sud, 65000 Tarbes, France

**Keywords:** Taekwondo, Postural control, Sprint running, Vertical jump, Pre-pubertal male

## Abstract

**Background:**

The postural and neuromuscular performances in healthy children taekwondo (TKD) practitioners in comparison with control children were examined.

**Methods:**

Seventeen healthy pre-pubertal males undertaking only physical education at school (age: 11.88 ± 0.33 years) and 12 pre-pubertal male TKD practitioners (>3 years, 4 sessions a week) (age 11.66 ± 0.49 years) were recruited. Performances in the dynamic postural control (Star Excursion Balance Test -SEBT), vertical jump [squat jump (SJ) and countermovement jump (CMJ)] and sprint running (distances: 5, 10, 20 and 30 m) tests were compared between the two groups.

**Results:**

The performances of the TKD practitioners were better than those of the non-TKD active for the SEBT (for 14 of 16 conditions, *p* < 0.05), SJ (*p* < 0.01), CMJ (*p* < 0.03) sprint running (5 m, *p* < 0.01; 10 m, *p* < 0.04; the performances for the 20 and 30 m sprints were not significant, *p* > 0.05).

**Conclusions:**

TKD practice would stimulate sensory input and motor output of the postural system that would enhance its efficiency. In addition, the dynamic nature of TKD would develop the muscle power of the lower limbs. In our sample of healthy pre-pubertal males, TKD appears to improve postural and neuromuscular functions, but further research is required.

**Electronic supplementary material:**

The online version of this article (doi:10.1186/s13102-016-0040-2) contains supplementary material, which is available to authorized users.

## Background

Motor experiences facilitate the maturation of the central nervous system in children, refining their postural and motor skills [1]. Moreover, certain physical and/or sport activities stimulate the postural and motor functions more than others. To improve these functions in an optimal way, physical and/or sport activity requires be completed by trying to react very quickly to a signal, to develop a muscle power or strength, to perform fast and well-coordinated motors skill and/or to perform technique movements in difficult postural conditions e.g. on monopodal dynamic stance.

Taekwondo (TKD) stimulates these motor abilities [2]. TKD focuses on kicking techniques. Rotation of the body and pivoting on one leg is an essential component in all of these kicking skills [3]. Elite TKD athletes turn and kick at high speeds (5.2 m/s to 16.26 m/s), and produce high striking forces (390.7 to 661.9 N) without losing balance [4]. The ability to perform fast and well-coordinated attack and defense actions are determining factors in TKD performance. To complete fast and powerful kicks, TKD practitioners require high muscle power and speed for kicking, and great dynamic postural control on the supporting leg. Therefore, the neuromuscular and postural abilities are determining factors for the athletes’ performance in competitions.

TKD training can improve knee and calf muscle strength in athletes at different levels of training [5–7]. Heller et al. [6] reported that the leg muscle powers are above normal in elite TKD athletes. Elite semiprofessional TKD athletes also demonstrate greater knee muscle strength than TKD novices. Recreational athletes may also benefit from TKD training in terms of neuromuscular qualities [8].

Few studies have addressed the effect of TKD training on postural control performance [9–13]. Noorul et al. [8] showed that, even with low-level TKD training (less than 4 h per week), recreational TKD practitioners had better balance performance than their sedentary counterparts when standing with their eyes closed after dropping from a height. TKD practitioners might even develop sport-specific balance ability [9, 14]. Seventeen weeks of TKD training could improve the balance time in single leg stance in elderly subjects [12]. Similarly, TKD training produced positive effects on balance and mobility in older adults, as indicated by the improvement in functional reach distance, Timed Up-and-Go test, walking velocity, and gait stability [11].

These two studies consistently showed that older adults could benefit from TKD training in terms of various balance components. To date, few studies have investigated the effects of TKD training in young healthy adolescents [9, 10, 14]. A study has shown that adolescents undertaking TKD training may have better balance performance than untrained subjects [9].

However, the effects of TKD training on the postural and neuromuscular functions were separately studied in healthy adults and adolescents, which does not allow the establishing of global effects of their functional abilities relative to posture and movement. In addition, these effects were not studied in healthy pre-pubertal children. Yet, with the increasing popularity of this sport and as many practitioners start training at a very young age [15], there is a need to examine the effect of TKD to ascertain whether TKD induces improvements in postural and neuromuscular functions in pre-pubertal healthy children. Hence, the aim of this study is to examine the postural and neuromuscular performances in healthy pre-pubertal male TKD practitioners in comparison to control males. It was hypothesized that the postural and neuromuscular performances of healthy pre-pubertal male TKD practitioners would perform significantly better in these tests than their non-TKD practicing counterpart.

## Methods

All the procedures described in the present investigations were approved by the Ethics Committee of the University of Manouba, Tunisia.

Signed written consent to publish was obtained from the children’s parent or legal guardian.

### Participants

Our population consisted of 17 pre-pubertal healthy males (age: 11.88 ± 0.33 years) taking only part in physical education lessons at school, for at least 3 years (non sport children), and 12 pre-pubertal healthy male TKD practitioners (age 11.66 ± 0.49 years) who had practiced TKD for 3.33 ± 0.49 years for four sessions a week (1 h30 per session). All the subjects were at Tanner’s stage 1 of puberty [16], with no differences in anthropometric characteristics between the 2 groups (Table 1). None of the subjects reported a history of hip, knee or ankle injury, or reported known pathological condition of the lower limb. All practitioners were right leg dominant, as ascertained by asking them which leg they preferred to kick a football with. All procedures were approved by Manouba University Institutional Review Committee. Written informed consent was obtained from all participants and their guiardian/parent after they had received both verbal and a written explanations of the experimental protocol and its potential risks and benefits. All participants and their parents were assured that they could withdraw from the trial without penalty at any time.

**Table 1:** Comparison of anthropometric measures (means ± standard deviations) between the non-athletic children and the taekwondo practitioners children. The significance level was set at *P* <0.05 (NS = non significant)

	Non sport children	TKD practitioners children	*P*
Height (cm)	148.31 ± 3.93	145.42 ± 3.75	NS
Leg length (cm)	79.83 ± 3.54	82.29 ± 3.180	NS
Body mass (kg)	42.52 ± 11.10	36.93 ± 5.11	NS

### Procedures

The experiment consisted in examining the anthropometric characteristics and the postural and neuromuscular (vertical jumps and sprint running) abilities for the two groups of subjects. Postural tests, vertical jump, sprint run tests, were administered over three different days, with one day of recovery between each day (i.e. between the postural control test, the vertical jump tests, and the sprint test) to avoid possible fatigue effects. All the tests were completed between 3 and 6 pm.

### Measures

#### Height and body mass

Subject’s height was measured (in cm) using a graduated and non-deformable measuring rod and their weight [body mass (BM) in kg] was evaluated using the EKS apparatus (Focus 9800, Sweden), without clothes and barefoot.

#### Leg length

Leg length of both legs were measured (in cm) with the subjects lying supine on an examination couch using a standard tape measurer from the anterior superior iliac spine to the distal end of the medial malleolus [17].

#### Dynamic Postural Control (DPC) test

**Fig. 1:**
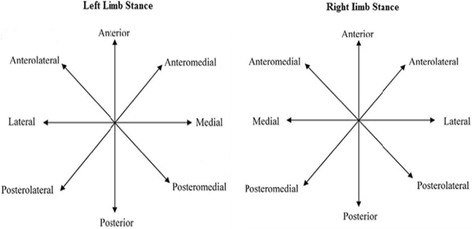
The 8 positions of the Star Excursion Balance Test are based on the stance limb

The Star Excursion Balance Test (SEBT) measured DPC. This functional, unilateral balance test integrates a single-leg stance with maximum reach of the opposite leg [18] The SEBT was performed with the subjects standing in the middle of a grid placed on the floor with 8 lines extending at 45° increments from the center of the grid. The 8 lines on the grid were named in relation to the direction of reach with regard to the stance leg: Anterolateral (AL), Anterior (A), Anteromedial (AM), Medial (M), posteromedial (PM), Posterior (P), Posterolateral (PL), and Lateral (L) (Fig. 1).

The protocol by Hertel et al. [18] was followed and the reach distances were normalized by dividing each excursion distance (in cm) by the subject’s leg length (in cm) and then multiplying the value obtained by 100.

#### Vertical jump tests

After a 10 min warm up (including progressive running exercises and stretching exercises), jumping height was assessed using an infrared photocell mat connected to a digital computer (Optojump System, Microgate SARL, Bolzano, Italy) by the same investigator. The optical acquisition system allowed the measurement of the contact time (t_c_) and the flight time (t_f_) during a jump, and calculated the height of the jump (h) to a precision of 1/1000s [19]. The height is measured using the equation as follows: h(cm) = g (t_f_) 2/8 (g : gravity).

For the squat jump (SJ) tests, the subjects started in the semi squat position with a knee flexion angle of nearly 90° and without moving, and the subjects had their hands placed on their hips. At the signal, the subject pushed off.

Countermovement jump (CMJ) tests started from an upright standing position with the subjects’ hands on their hips. At the signal, the subjects made a downward movement until reaching an approximate knee angle of 90°, and subsequently began to push-off. All subjects performed familiarization trials before undertaking three consecutive experimental trials for the two tests (SJ and CMJ). The highest value for each jump (test) was retained. Every subject was given a 15-s interval between attempts.

#### Sprint run (30 m)

After a 15 min warm-up, the subject ran at his maximum speed a distance of 30 m from a standing start. Photoelectric cells (radio system, Microgate, Bolzano, Italy) were placed at 5, 10, 20 and 30 m. Each subject took the test three times with an interval of 5 min of recovery after each test. The best value (in s) for each distance was used for statistical analyses.

### Analysis

Statistical analyses were performed using SPSS for Windows software (version 17.0). The descriptive values are presented as mean and standard deviation. The reproducibility of measurements of DPC, SJ, CMJ and 30 m sprint were determined by calculating the interclass coefficient of correlation (ICC). The estimation of this coefficient was based on a process of analysis of variance (ANOVA): an ICC from 0.80 to 1.00 is considered very reproducible, 0.60 to 0.79 moderately reproducible, and less than 0.60 representing a questionable reproducibility. Before using parametric statistics, it was checked the normality of the test variables Shapiro-Wilk W. The independent *t* Test was used to detect any differences between two groups. Cohen’s effect sizes were also calculated using GPOWER software (Bonn FRG, Bonn University, Department of Psychology) [20]. The results were interpreted using the following criteria: 0.20 [small]; 0.50 [medium]; 0.80 [large] [21]. Significance level was set at *p* < 0.05.

## Results

The interclass correlation coefficients on the reproducibility (ICCs) showed an excellent reliability for the DPC test concerning the right and the left supporting legs and for the SJ, CMJ and sprint tests (Table 2).

**Table 2:** The coefficient of intraclass correlation on the reproducibility of vertical jumps, speed run and dynamic postural control tests

			ICC	95 % IC
Vertical jumps (cm)	SJ		0.952	0.981–0.983
	CMJ		0.961	0.911–0.985
Speed run (s)	5 m		0.883	0.624–0.934
	10 m		0.929	0.841–0.972
	20 m		0.964	0.919–0.986
	30 m		0.967	0.926–0.987
Dynamic postural control (cm)	Anterolateral	Right	0.994	0.988–0.997
		Left	0.997	0.994–0.998
	Anterior	Right	0.996	0.993–0.998
		Left	0.990	0.982–0.995
	Anteromedial	Right	0.993	0.988–0.997
		Left	0.995	0.991–0.997
	Medial	Right	0.997	0.995–0.999
		Left	0.998	0.996–0.999
	Posteromedial	Right	0.995	0.991–0.998
		Left	0.997	0.994–0.998
	Posterior	Right	0.997	0.994–0.998
		Left	0.996	0.992–0.998
	Posterolateral	Right	0.997	0.994–0.998
		Left	0.997	0.995–0.999
	Lateral	Right	0.997	0.994–0.998
		Left	0.993	0.987–0.996

*ICC* interclass correlation coefficients, *CI* Confidence interval

Most of the results on postural data were significantly different between the non-athletic practitioners and the TKD practitioners. Fourteen of 16 measures were significantly different between the two groups (Table 3).

**Table 3:** Comparison of values (means ± standard deviations) and effect size of vertical jumps, speed run and dynamic postural control between the non sport children and the TKD practitioners children. The significance level was set at *P* <0.05 (NS = non significant)

			Non sport children	TKD practitioners children	Effect size	*P*
Vertical jumps (cm)	SJ		18.85 ± 4.00	24.30 ± 4.85	1.22 [large]	0.003
	CMJ		19.54 ± 4.23	23.53 ± 5.44	0.81 [large]	0.035
Speed run (s)	5 m		1.30 ± 0.10	1.54 ± 0.33	0.98 [large]	0.008
	10 m		2.23 ± 0.16	2.43 ± 0.30	0.83 [large]	0.038
	20 m		3.98 ± 0.30	4.06 ± 0.31	0.26 [medium]	NS
	30 m		5.72 ± 0.41	5.74 ± 0.38	0.05 [small]	NS
Dynamic postural control (cm)	Anterolateral	Right	78.4 ± 10.8	83.5 ± 6.6	0.56 [large]	NS
		Left	77.2 ± 10.6	93.8 ± 7.5	1.80 [large]	0.000
	Anterior	Right	79.4 ± 9.4	83.9 ± 1.01	0.67 [large]	NS
		Left	79.6 ± 8.7	86.1 ± 5.4	0.59 [large]	0.031
	Anteromedial	Right	83.5 ± 1.08	91.5 ± 8.4	1.33 [large]	0.042
		Left	82.8 ± 8.9	94.0 ± 7.6	1.35 [large]	0.002
	Medial	Right	80.7 ± 11.4	93.1 ± 9.8	1.16 [large]	0.005
		Left	80.04 ± 10.0	96,3 ± 9,5	1.67 [large]	0.000
	Posteromedial	Right	85.1 ± 1.48	95.9 ± 9.2	1.63 [large]	0.034
		Left	81.7 ± 9.2	92.1 ± 6.9	1.27 [large]	0.003
	Posterior	Right	73.2 ± 14.7	94.4 ± 9.01	1.73 [large]	0.000
		Left	74.9 ± 12.8	94.1 ± 7.6	1.82 [large]	0.000
	Posterolateral	Right	69.1 ± 1.04	87.1 ± 1.1	1.68 [large]	0.000
		Left	71.9 ± 1.1	94.2 ± 7.8	4.00 [large]	0.000
	Lateral	Right	61.2 ± 12.3	79.4 ± 9.9	1.63 [large]	0.000
		Left	65.66 ± 10.3	93.5 ± 7.4	3.10 [large]	0.000

The performances in vertical jumps (SJ and CMJ) were significantly different between the non-athletic practitioners and the TKD practitioners (Table 3).

The sprint performances differed between the two groups only for the very short distances (5 and 10 m). The performances for the other distances (20 and 30 m) did not differ between the non-sport and TKD practitioners (Table 3).

All the raw data are available in Additional files 1, 2, 3, and 4 enclosed.

## Discussion

The present investigation showed that the performances in dynamic postural control, vertical jumps (SJ and CMJ) and very short sprint (5 and 10 m) were significantly better for the TKD practitioners than for the non-athletic males.

The fact that the performance in the DPC test was better for the TKD practitioners than for the non-athletic children is not surprising. First, in general, regular sport practice improves the postural system output [22] by refining its sensory and motor functions as well as its central integration of sensory information [23–26]. Moreover, in children, motor experience facilitates the building of a repertoire of postural strategies and enables the child to select the most appropriate postural strategy, depending on the ability to anticipate the consequence of the movement to maintain balance control and optimal efficiency of the motor task [1]. The DPC test aims to reach the maximal distance with the opposite leg while on monopodal stance. The movements involved by this test are destabilizing, and require anticipation of the consequence of movement. This could mean that TKD practice can favorably influence the ability to anticipate the consequence of the movement in children.

Previous studies have shown that TKD practice improves postural control in adolescents, adults, older subjects [9, 10, 27] and in children with a variety of pathologies [5]. However, no study has shown that TKD can enhance postural abilities in healthy children. Obviously, TKD requires dynamic stability on the supporting leg (e.g. rotation of the body and pivoting on one leg) to perform fast, ballistic movements by the kicking leg aimed to hit the opponent [14]. To maintain balance in this context, the TKD practitioner children strongly stimulate their different sensory receptors, particularly proprioceptive, vestibular, visual and cutaneous receptors. Repetitive and regular stimulations of these receptors related to TKD practice would refine their sensitivity. This would explain why young TKD practitioners exhibited more efficient somatosensory and vestibular inputs than untrained children [9, 10, 27]. In addition, learning of technique movements during the practice of combat sports can influence postural adaptation by motor program acquisitions that include specific postural adaptations [28]. As the postural task of DPC (i.e. moving one leg by supporting the body on the other leg) is relatively close to that observed for kicking techniques in TKD (i.e. performing a kicking technique while supporting on one leg ony), one could hypothesize that specific postural adaptations could occur in healthy children. The motor function of the postural system involving movement control and command could benefit specific postural adaptations. Fong et al.’ study [29] reinforces this hypothesis, since it reported that TKD practitioners seem to exhibit sport-specific balance ability. These authors demonstrated that adolescent TKD practitioners required less time to complete a 180°-turn and swayed less during turning than non-practitioners. This hypothesis can be also corroborated through neurophysiological considerations since Chung and Ng [30] showed that professional TKD practitioners have better neuromotor ability than non-athletes in both large and small muscles, with faster reactions to sport-specific stimuli, suggesting a generalized motor experience effect across various muscles.

In addition, better neuromotor ability could explain why the postural performances for both dominant and non-dominant supporting legs were better for the TKD practitioners than for the non-sporting children. However, two of 16 postural conditions were non significant for the dominant supporting leg while all the conditions were significant for the non-dominant leg. The specific postural adaptations would be thus more marked for the non-dominant supporting leg than for the dominant supporting leg. This phenomenon would be linked to the fact that kicking techniques are more frequently performed on the non-dominant supporting leg than on the dominant supporting leg in TKD practitioners. Hence, the solicitation time was longer on the non-supporting leg than on the dominant leg and thus would induce further postural adaptations.

TKD practitioners exhibited better performance in vertical jump with or without downward movement (SJ and CMJ). This may mean that the muscle power and myotendineous elasticity were greater for the young TKD practitioners than for the age matched non-athletic children [31]. Power is the product of force by velocity: our results represent either a greater force or a greater speed, or both, in favour of the TKD practitioners. Thus, TKD practice could induce either an improvement of muscle strength through better spatial and temporal recruitment of motor units, or an improvement in the speed of muscle action through better synchronisation of motor units firing during the vertical jump test [32]. As the anthropometric characteristics of the young TKD practitioners did not differ from those of the non-athletic children, they were unlikely to influence the performance in vertical jump. Hence, the best performance in vertical jump for the practitioners TKD children would be related to better intrinsic qualities of the neuromuscular function. Indeed, improvements in neuromuscular performance can be observed with or without increase in muscle mass [33]. Moreover, multiple and repetitive skipping and jumps during TKD practice would stimulate the myotendineous structures of the lower limb, and would thus enhance the myotendineous elasticity in healthy children.

The fact that TKD children had greater muscle power than the non-athletic children could explain why they were better in short sprint run (5 and 10 m) i.e. for distances whose power abilities are determining in terms of performance [34]. The children practicing TKD were not faster than the non-athletic children over 20 and 30 m. This is not surprising, as specificity of sport would suggest that, as TKD is based on extremely fast accelerations over very short distances, the experience effect in TKD would manifest itself only over the shorter sprint distances [34]. To analyze more precisely the locomotor effects induced by the TKD practice, future studies could use gait analysis to investigate the biomechanics of sprint running in relation to stride length and stride rate over the different distances considered.

This study presents limitations: for example, it did not evaluate the effects of TKD training but only compared the postural and neuromuscular performances between healthy pre-pubertal males TKD practitioners and healthy non-athletic males of the same age. The study did show that young male TKD practitioners were more performant than the non-practitioner children, but only a longitudinal study would determine whether TKD training improves performance in these performance variables.

## Conclusions

The performances in postural control, vertical jumps and very short sprint running were significantly better in healthy pre-pubertal males TKD practitioners than for non-athletic males of the same age. TKD practice would stimulate sensory input and motor output of the postural system, which would improve its efficiency particularly in specific postural conditions, i.e. on one leg support. In addition, the dynamic nature of TKD could develop the power of the muscles of the lower limb. TKD training is likely to facilitate postural and neuromuscular functions in healthy children.

## Additional files

Additional file 1: Vertical Jump NO SPORTS. (DOC 46 kb) Additional file 2: Dynamic Postural Control NO SPORTS. (DOC 116 kb) Additional file 3: Dynamic Postural Control (TKD). (DOC 87 kb) Additional file 4: SPEED NO SPORTS. (DOC 47 kb)

## Abbreviations

A: anterior
AL: anterolateral
AM: anteromedial
BM: body mass
CMJ: countermovement jump
DPC: dynamic postural control
L: lateral
M: medial
P: posterior
PL: posterolateral
PM: posteromedial
SEBT: Star Excursion Balance Test
SJ: squat jump
TKD: Taekwondo
