# Supplementary material for: Pre-pubertal males practising Taekwondo exhibit favourable postural and neuromuscular performance
Source: BMC Sports Sci Med Rehabil. 2016 Jun 4;8:16. doi: 10.1186/s13102-016-0040-2 (PMC4893255; doi:10.1186/s13102-016-0040-2)
Supplement: Additional file 1: — Vertical Jump NO SPORTS. (DOC 46 kb) [file 13102_2016_40_MOESM1_ESM.doc]

| **Name and Fisrt Name** | **SJ** | | | **CMJ** | | |
| --- | --- | --- | --- | --- | --- | --- |
| Iheb bouselmi | 26.6 | 20.7 | 23.1 | 17.7 | 19.4 | 21.2 |
| Med Feres Dridi | 18.2 | 16.6 | 169.5 | 16 | 22.5 | 22.5 |
| Oussema Dhahri | 15.5 | 15.9 | 15.4 | 17.1 | 15.4 | 14.4 |
| Med Amine Hajji | 20 | 18.2 | 18.2 | 18.8 | 20 | 20 |
| Med Abbes Jbéli | 21.9 | 18.2 | 20.7 | 21.9 | 21.3 | 21.9 |
| Badis Daaji | 17 | 16.5 | 13.4 | 15.9 | 15.4 | 16.5 |
| Bechir Hosni | 12 | 12.8 | 12 | 13.9 | 12.4 | 11.9 |
| Akrem Aouini | 19.5 | 20 | 15.4 | 18.8 | 22.5 | 18.8 |
| Nabil Beji | 21.3 | 21.9 | 19.4 | 25.2 | 25.1 | 23.8 |
| Rayen Rafrafi | 21.3 | 20.7 | 22.5 | 23.1 | 24.4 | 23.1 |
| Med Amine Boudabbous | 19.4 | 20 | 18.8 | 19.5 | 19.4 | 20 |
| Saif Rourou | 23.8 | 25.8 | 23.1 | 23.1 | 25.8 | 25.9 |
| Med Malek Teji | 10.5 | 10.5 | 11.9 | 8.8 | 10.1 | 11.9 |
| Med Iheb Marsaoui | 14.9 | 12.9 | 13.4 | 14.9 | 13.9 | 13.4 |
| Ala Weraghni | 15.4 | 16.0 | 17.1 | 14.9 | 15.4 | 16.6 |
| Hassene Makni | 15.9 | 15.9 | 17.1 | 15.9 | 17.1 | 16.5 |
| Med Amine Aloui | 17.1 | 16.6 | 18.2 | 18.2 | 17.1 | 18.2 |

**Vertical Jump NO SPORTS**
