# Supplementary material for: Pre-pubertal males practising Taekwondo exhibit favourable postural and neuromuscular performance
Source: BMC Sports Sci Med Rehabil. 2016 Jun 4;8:16. doi: 10.1186/s13102-016-0040-2 (PMC4893255; doi:10.1186/s13102-016-0040-2)
Supplement: Additional file 2: — Dynamic Postural Control NO SPORTS. (DOC 116 kb) [file 13102_2016_40_MOESM2_ESM.doc]

**Dynamic Postural Control NO SPORTS**

**Left leg on the ground**

| Name and Fisrt Name | Length  Member  Inferior (cm) | Mass  (Kg) | Height (cm) | **Results obtained in each axis**  **(cm)** | | | | | | | | | | | | | | | | | | | | | | | |
| --- | --- | --- | --- | --- | --- | --- | --- | --- | --- | --- | --- | --- | --- | --- | --- | --- | --- | --- | --- | --- | --- | --- | --- | --- | --- | --- | --- |
|  |  |  |  | Axis (a) | | | Axis (b) | | | Axis (c) | | | Axis (d) | | | Axis (e) | | | Axis (f) | | | Axis (g) | | | Axis (h) | | |
| Iheb bouselmi | 80 | 55.8 | 152 | 75 | 75 | 76 | 70 | 69 | 71 | 70 | 71 | 72 | 75 | 76 | 75 | 70 | 71 | 72 | 72 | 71 | 72 | 60 | 62 | 61 | 69 | 70 | 71 |
| Med Feres Dridi | 80.5 | 45.8 | 154.2 | 70 | 67 | 70 | 70 | 71 | 72 | 70 | 71 | 71 | 70 | 71 | 70 | 61 | 63 | 64 | 60 | 60 | 61 | 50 | 51 | 50 | 65 | 64 | 65 |
| Oussema Dhahri | 81.5 | 30.4 | 148.5 | 62 | 62 | 61 | 60 | 61 | 62 | 60 | 61 | 63 | 70 | 69 | 71 | 50 | 52 | 53 | 50 | 53 | 54 | 50 | 49 | 51 | 59 | 60 | 61 |
| Med Amine Hajji | 78.5 | 40.8 | 148.5 | 60 | 61 | 63 | 65 | 64 | 65 | 67 | 69 | 69 | 60 | 59 | 62 | 59 | 60 | 61 | 55 | 56 | 60 | 45 | 46 | 50 | 60 | 61 | 60 |
| Med Abbes Jbéli | 81 | 38.5 | 158.5 | 70 | 71 | 70 | 75 | 76 | 77 | 80 | 81 | 82 | 75 | 74 | 75 | 80 | 81 | 80 | 70 | 71 | 70 | 70 | 71 | 70 | 70 | 72 | 71 |
| Badis Daaji | 73.5 | 32.4 | 139.5 | 55 | 56 | 56 | 56 | 57 | 60 | 60 | 63 | 64 | 64 | 62 | 64 | 55 | 56 | 57 | 55 | 56 | 57 | 45 | 49 | 50 | 52 | 52 | 53 |
| Bechir Hosni | 83.5 | 52 | 160.1 | 63 | 65 | 64 | 67 | 69 | 67 | 60 | 59 | 60 | 60 | 59 | 60 | 50 | 52 | 50 | 52 | 50 | 53 | 52 | 54 | 55 | 70 | 69 | 70 |
| Akrem Aouini | 80 | 44.5 | 152.1 | 60 | 61 | 60 | 65 | 64 | 65 | 60 | 61 | 60 | 60 | 62 | 63 | 60 | 61 | 62 | 50 | 52 | 50 | 52 | 54 | 52 | 55 | 54 | 55 |
| Nabil Beji | 80.4 | 39.4 | 149.3 | 60 | 61 | 60 | 65 | 66 | 67 | 60 | 61 | 60 | 62 | 63 | 64 | 57 | 59 | 60 | 64 | 65 | 66 | 50 | 51 | 50 | 54 | 53 | 54 |
| Rayen Rafrafi | 74 | 31.5 | 142.5 | 64 | 65 | 64 | 60 | 61 | 60 | 60 | 62 | 60 | 69 | 67 | 67 | 60 | 59 | 60 | 50 | 51 | 50 | 50 | 52 | 54 | 55 | 57 | 60 |
| Med Amine Boudabbous | 85.2 | 46.4 | 156 | 70 | 72 | 73 | 80 | 81 | 80 | 75 | 74 | 75 | 75 | 74 | 75 | 70 | 72 | 70 | 63 | 64 | 65 | 50 | 55 | 57 | 75 | 74 | 75 |
| Saif Rourou | 80.5 | 40.3 | 153 | 65 | 64 | 65 | 67 | 68 | 69 | 65 | 64 | 65 | 64 | 63 | 64 | 65 | 64 | 65 | 65 | 64 | 65 | 60 | 71 | 60 | 65 | 65 | 66 |
| Med Malek Chebbi | 82 | 73.9 | 154 | 60 | 59 | 60 | 60 | 61 | 60 | 60 | 59 | 60 | 50 | 50 | 51 | 43 | 42 | 44 | 50 | 50 | 50 | 40 | 45 | 45 | 60 | 59 | 60 |
| Med Iheb Marsaoui | 83 | 48.5 | 153 | 52 | 53 | 53 | 60 | 59 | 60 | 55 | 54 | 55 | 55 | 55 | 55 | 45 | 45 | 46 | 37 | 38 | 39 | 40 | 42 | 40 | 50 | 51 | 50 |
| Ala Weraghni | 83 | 39.6 | 151.5 | 65 | 65 | 64 | 70 | 69 | 71 | 62 | 63 | 64 | 70 | 69 | 70 | 70 | 71 | 70 | 60 | 61 | 62 | 50 | 55 | 57 | 65 | 64 | 66 |
| Hassene Makni | 73 | 33 | 139.5 | 55 | 55 | 56 | 55 | 57 | 59 | 50 | 50 | 51 | 60 | 61 | 63 | 52 | 51 | 53 | 47 | 48 | 49 | 40 | 40 | 43 | 49 | 48 | 49 |
| Med Amine Aloui | 77.5 | 30 | 148.5 | 75 | 74 | 76 | 74 | 75 | 75 | 70 | 71 | 70 | 70 | 71 | 70 | 65 | 66 | 67 | 60 | 64 | 65 | 60 | 61 | 62 | 63 | 64 | 63 |

**Right leg on the ground**

| Name and Fisrt Name | Length  Member  Inferior (cm) | Mass  (Kg) | Height (cm) | **Results obtained in each axis**  **(cm)** | | | | | | | | | | | | | | | | | | | | | | | |
| --- | --- | --- | --- | --- | --- | --- | --- | --- | --- | --- | --- | --- | --- | --- | --- | --- | --- | --- | --- | --- | --- | --- | --- | --- | --- | --- | --- |
|  |  |  |  | Axis (a) | | | Axis (b) | | | Axis (c) | | | Axis (d) | | | Axis (e) | | | Axis (f) | | | Axis (g) | | | Axis (h) | | |
| Iheb bouselmi | 80 | 55.8 | 152 | 65 | 66 | 66 | 75 | 75 | 77 | 75 | 75 | 76 | 85 | 86 | 88 | 78 | 79 | 80 | 60 | 60 | 61 | 40 | 45 | 47 | 75 | 76 | 77 |
| Med Feres Dridi | 80.5 | 45.8 | 154.2 | 70 | 71 | 70 | 78 | 80 | 81 | 73 | 72 | 73 | 78 | 80 | 81 | 65 | 67 | 69 | 64 | 65 | 66 | 45 | 46 | 47 | 70 | 71 | 70 |
| Oussema Dhahri | 81.5 | 30.4 | 148.5 | 67 | 68 | 69 | 70 | 69 | 71 | 55 | 56 | 57 | 70 | 71 | 70 | 50 | 51 | 50 | 44 | 43 | 45 | 45 | 44 | 45 | 55 | 57 | 59 |
| Med Amine Hajji | 78.5 | 40.8 | 148.5 | 50 | 50 | 51 | 55 | 57 | 59 | 59 | 60 | 60 | 63 | 65 | 67 | 52 | 53 | 54 | 45 | 46 | 47 | 35 | 37 | 39 | 51 | 52 | 50 |
| Med Abbes Jbéli | 81 | 38.5 | 158.5 | 70 | 71 | 71 | 72 | 73 | 73 | 85 | 85 | 85 | 90 | 90 | 91 | 81 | 82 | 82 | 65 | 66 | 66 | 70 | 69 | 70 | 70 | 71 | 70 |
| Badis Daaji | 73.5 | 32.4 | 139.5 | 50 | 50 | 50 | 49 | 49 | 50 | 50 | 50 | 50 | 55 | 56 | 55 | 40 | 41 | 42 | 51 | 51 | 52 | 44 | 43 | 45 | 55 | 52 | 52 |
| Bechir Hosni | 83.5 | 52 | 160.1 | 60 | 61 | 62 | 65 | 66 | 65 | 60 | 59 | 61 | 54 | 55 | 56 | 45 | 46 | 47 | 52 | 53 | 52 | 42 | 45 | 45 | 59 | 60 | 60 |
| Akrem Aouini | 80 | 44.5 | 152.1 | 60 | 61 | 60 | 61 | 64 | 62 | 60 | 61 | 60 | 66 | 67 | 70 | 60 | 62 | 64 | 50 | 52 | 53 | 42 | 43 | 44 | 64 | 65 | 65 |
| Nabil Beji | 80.4 | 39.4 | 149.3 | 60 | 61 | 60 | 65 | 67 | 69 | 70 | 71 | 71 | 70 | 72 | 73 | 60 | 61 | 62 | 50 | 51 | 52 | 50 | 49 | 50 | 65 | 67 | 67 |
| Rayen Rafrafi | 74 | 31.5 | 142.5 | 60 | 61 | 60 | 65 | 64 | 67 | 62 | 63 | 64 | 69 | 67 | 67 | 50 | 52 | 51 | 50 | 51 | 50 | 40 | 41 | 40 | 50 | 55 | 54 |
| Med Amine Boudabbous | 85.2 | 46.4 | 156 | 75 | 76 | 75 | 80 | 81 | 82 | 70 | 71 | 72 | 75 | 72 | 71 | 62 | 61 | 63 | 65 | 66 | 65 | 60 | 61 | 62 | 80 | 81 | 82 |
| Saif Rourou | 80.5 | 40.3 | 153 | 65 | 66 | 66 | 65 | 67 | 67 | 65 | 64 | 65 | 70 | 70 | 70 | 60 | 64 | 63 | 65 | 64 | 65 | 60 | 61 | 60 | 65 | 66 | 6565 |
| Med Malek Chebbi | 82 | 73.9 | 154 | 65 | 64 | 65 | 60 | 64 | 62 | 55 | 55 | 56 | 49 | 50 | 50 | 45 | 46 | 45 | 50 | 49 | 50 | 40 | 40 | 40 | 55 | 55 | 54 |
| Med Iheb Marsaoui | 83 | 48.5 | 153 | 55 | 55 | 55 | 60 | 61 | 60 | 60 | 61 | 60 | 65 | 65 | 65 | 50 | 50 | 51 | 50 | 49 | 50 | 45 | 45 | 46 | 55 | 56 | 56 |
| Ala Weraghni | 83 | 39.6 | 151.5 | 64 | 65 | 64 | 64 | 63 | 64 | 60 | 61 | 62 | 63 | 62 | 64 | 60 | 60 | 61 | 60 | 61 | 60 | 60 | 59 | 60 | 65 | 64 | 65 |
| Hassene Makni | 73 | 33 | 139.5 | 60 | 61 | 60 | 59 | 60 | 61 | 57 | 59 | 60 | 50 | 52 | 54 | 50 | 49 | 52 | 45 | 45 | 45 | 40 | 40 | 41 | 57 | 60 | 61 |
| Med Amine Aloui | 77.5 | 30 | 148.5 | 67 | 68 | 68 | 68 | 67 | 68 | 60 | 62 | 61 | 66 | 67 | 67 | 54 | 53 | 54 | 65 | 64 | 65 | 60 | 60 | 60 | 69 | 69 | 67 |
