# Supplementary material for: Pre-pubertal males practising Taekwondo exhibit favourable postural and neuromuscular performance
Source: BMC Sports Sci Med Rehabil. 2016 Jun 4;8:16. doi: 10.1186/s13102-016-0040-2 (PMC4893255; doi:10.1186/s13102-016-0040-2)
Supplement: Additional file 3: — Dynamic Postural Control (TKD). (DOC 87 kb) [file 13102_2016_40_MOESM3_ESM.doc]

**Dynamic Postural Control (TKD)**

**Right leg on the ground**

| Name and Fisrt Name | Length  Member  Inferior (cm) | Mass  (Kg) | Height (cm) | Years of practice  TKD  (years) | **Results obtained in each axis**  **(cm)** | | | | | | | | | | | | | | | | | | | | | | | |
| --- | --- | --- | --- | --- | --- | --- | --- | --- | --- | --- | --- | --- | --- | --- | --- | --- | --- | --- | --- | --- | --- | --- | --- | --- | --- | --- | --- | --- |
|  |  |  |  |  | Axis (a) | | | Axis (b) | | | Axis (c) | | | Axis (d) | | | Axis (e) | | | Axis (f) | | | Axis (g) | | | Axis (h) | | |
| Med Wassim Jlassi | 79 | 31.1 | 139 | 4 | 50 | 51 | 52 | 60 | 61 | 61 | 59 | 59 | 59 | 72 | 73 | 74 | 65 | 64 | 64 | 61 | 62 | 61 | 62 | 63 | 65 | 63 | 64 | 65 |
| Zied mathlouthi | 75.5 | 38.8 | 149 | 3 | 75 | 76 | 76 | 82 | 81 | 82 | 82 | 83 | 83 | 90 | 88 | 88 | 86 | 85 | 86 | 81 | 82 | 84 | 75 | 75 | 76 | 74 | 75 | 75 |
| Hakim ben Dhief | 87 | 34.9 | 147 | 3 | 61 | 62 | 62 | 66 | 70 | 72 | 75 | 76 | 78 | 75 | 74 | 75 | 67 | 70 | 72 | 62 | 64 | 66 | 50 | 52 | 52 | 68 | 70 | 68 |
| Saifeddine Bougdida | 80 | 33.4 | 143 | 3 | 70 | 71 | 72 | 80 | 82 | 82 | 88 | 90 | 88 | 83 | 85 | 87 | 80 | 81 | 80 | 80 | 81 | 80 | 73 | 75 | 75 | 68 | 70 | 70 |
| Safouene Zouaoui | 82 | 32.8 | 141 | 3 | 68 | 70 | 70 | 70 | 75 | 75 | 73 | 75 | 75 | 70 | 72 | 72 | 80 | 81 | 80 | 70 | 71 | 70 | 61 | 62 | 64 | 63 | 64 | 65 |
| Azer Nasser | 86 | 35.8 | 147.5 | 4 | 70 | 71 | 70 | 74 | 75 | 74 | 83 | 84 | 85 | 90 | 90 | 90 | 80 | 81 | 80 | 73 | 73 | 74 | 70 | 71 | 70 | 60 | 63 | 63 |
| Amen Allah Troudi | 84.5 | 40.8 | 153 | 3 | 62 | 65 | 68 | 76 | 76 | 77 | 75 | 76 | 78 | 75 | 80 | 81 | 78 | 79 | 80 | 72 | 74 | 74 | 70 | 71 | 70 | 70 | 71 | 73 |
| Jasser Hmili | 83.5 | 38.3 | 147.5 | 3 | 70 | 71 | 70 | 75 | 78 | 78 | 80 | 79 | 77 | 70 | 70 | 69 | 75 | 73 | 76 | 62 | 65 | 65 | 59 | 60 | 59 | 75 | 75 | 75 |
| Amir Chichi | 81 | 50.3 | 144 | 2 | 70 | 71 | 70 | 71 | 74 | 75 | 70 | 71 | 72 | 75 | 76 | 74 | 62 | 64 | 67 | 60 | 61 | 62 | 52 | 53 | 54 | 60 | 61 | 62 |
| Youssef Ammar | 76 | 37.2 | 146 | 4 | 80 | 79 | 80 | 80 | 83 | 80 | 80 | 82 | 80 | 85 | 86 | 87 | 85 | 87 | 88 | 84 | 85 | 86 | 62 | 63 | 65 | 72 | 73 | 73 |
| Mohamed Fathallah | 81.5 | 32.5 | 144.5 | 2 | 72 | 73 | 73 | 79 | 80 | 81 | 79 | 80 | 80 | 80 | 80 | 80 | 80 | 80 | 80 | 70 | 73 | 74 | 70 | 71 | 70 | 70 | 71 | 70 |
| Adnene Khiari | 81.5 | 37.7 | 143.5 | 4 | 70 | 72 | 70 | 75 | 76 | 75 | 65 | 67 | 70 | 75 | 74 | 75 | 75 | 76 | 75 | 70 | 71 | 70 | 70 | 71 | 70 | 70 | 72 | 71 |

**Dynamic Postural Control (TKD)**

**Left leg on the ground**

| Name and Fisrt Name | Length  Member  Inferior (cm) | Mass  (Kg) | Height (cm) | Years of practice  TKD  (years) | **Results obtained in each axis**  **(cm)** | | | | | | | | | | | | | | | | | | | | | | | |
| --- | --- | --- | --- | --- | --- | --- | --- | --- | --- | --- | --- | --- | --- | --- | --- | --- | --- | --- | --- | --- | --- | --- | --- | --- | --- | --- | --- | --- |
|  |  |  |  |  | Axis (a) | | | Axis (b) | | | Axis (c) | | | Axis (d) | | | Axis (e) | | | Axis (f) | | | Axis (g) | | | Axis (h) | | |
| Med Wassim Jlassi | 79 | 31.1 | 139 | 4 | 70 | 70 | 70 | 80 | 80 | 80 | 70 | 70 | 72 | 73 | 75 | 74 | 80 | 81 | 80 | 70 | 69 | 70 | 55 | 56 | 55 | 70 | 70 | 71 |
| Zied mathlouthi | 75.5 | 38.8 | 149 | 3 | 710 | 72 | 70 | 80 | 82 | 80 | 85 | 85 | 84 | 85 | 85 | 84 | 80 | 81 | 80 | 802 | 80 | 81 | 70 | 71 | 70 | 74 | 73 | 74 |
| Hakim ben Dhief | 87 | 34.9 | 147 | 3 | 64 | 66 | 70 | 70 | 72 | 72 | 75 | 76 | 75 | 73 | 75 | 76 | 75 | 76 | 75 | 65 | 63 | 65 | 65 | 65 | 64 | 70 | 71 | 70 |
| Saifeddine Bougdida | 80 | 33.4 | 143 | 3 | 70 | 71 | 72 | 80 | 82 | 80 | 90 | 90 | 88 | 90 | 88 | 90 | 88 | 88 | 89 | 80 | 76 | 80 | 70 | 71 | 71 | 75 | 73 | 75 |
| Safouene Zouaoui | 82 | 32.8 | 141 | 3 | 70 | 70 | 70 | 72 | 73 | 72 | 73 | 74 | 74 | 80 | 82 | 82 | 80 | 78 | 80 | 65 | 65 | 66 | 64 | 64 | 65 | 65 | 64 | 65 |
| Azer Nasser | 86 | 35.8 | 147.5 | 4 | 80 | 81 | 79 | 90 | 90 | 91 | 94 | 91 | 93 | 95 | 95 | 95 | 90 | 90 | 90 | 82 | 82 | 81 | 70 | 71 | 70 | 73 | 73 | 74 |
| Amen Allah Troudi | 84.5 | 40.8 | 153 | 3 | 71 | 71 | 74 | 80 | 81 | 80 | 80 | 81 | 80 | 75 | 76 | 75 | 82 | 80 | 82 | 70 | 72 | 70 | 72 | 70 | 72 | 80 | 81 | 80 |
| Jasser Hmili | 83.5 | 38.3 | 147.5 | 3 | 70 | 73 | 70 | 75 | 76 | 78 | 80 | 79 | 80 | 85 | 84 | 84 | 84 | 85 | 84 | 75 | 74 | 75 | 61 | 60 | 61 | 75 | 74 | 75 |
| Amir Chichi | 81 | 50.3 | 144 | 2 | 64 | 62 | 63 | 69 | 70 | 71 | 70 | 70 | 70 | 75 | 76 | 76 | 62 | 64 | 65 | 55 | 56 | 55 | 50 | 50 | 50 | 70 | 71 | 70 |
| Youssef Ammar | 76 | 37.2 | 146 | 4 | 72 | 73 | 72 | 80 | 80 | 81 | 85 | 84 | 85 | 80 | 81 | 81 | 80 | 79 | 80 | 80 | 80 | 81 | 70 | 72 | 73 | 80 | 79 | 80 |
| Mohamed Fathallah | 81.5 | 32.5 | 144.5 | 2 | 70 | 71 | 70 | 73 | 70 | 73 | 80 | 79 | 80 | 78 | 80 | 82 | 81 | 82 | 82 | 80 | 81 | 80 | 70 | 71 | 70 | 69 | 70 | 69 |
| Adnene Khiari | 81.5 | 37.7 | 143.5 | 4 | 72 | 71 | 73 | 74 | 75 | 74 | 70 | 70 | 70 | 75 | 75 | 76 | 71 | 73 | 75 | 70 | 70 | 70 | 60 | 61 | 60 | 65 | 66 | 65 |
