# Supplementary material for: Pre-pubertal males practising Taekwondo exhibit favourable postural and neuromuscular performance
Source: BMC Sports Sci Med Rehabil. 2016 Jun 4;8:16. doi: 10.1186/s13102-016-0040-2 (PMC4893255; doi:10.1186/s13102-016-0040-2)
Supplement: Additional file 4: — SPEED NO SPORTS. (DOC 47 kb) [file 13102_2016_40_MOESM4_ESM.doc]

**SPEED NO SPORTS**

| **Name and Fisrt Name** | **5M**  **(S)** | | | **10M**  **(S)** | | | **20M**  **(S)** | | | **30M**  **(S)** | | |
| --- | --- | --- | --- | --- | --- | --- | --- | --- | --- | --- | --- | --- |
| Iheb bouselmi | 1.37 | 1.36 | 1.35 | 2.31 | 2.34 | 2.32 | 4.05 | 4.17 | 4.12 | 5.85 | 6.01 | 5.86 |
| Med Feres Dridi | 1.27 | 1.28 | 1.41 | 2.17 | 2.18 | 2.31 | 3.85 | 3.96 | 4.01 | 5.81 | 5.63 | 563 |
| Oussema Dhahri | 1.39 | 1.31 | 1.67 | 2.39 | 2.32 | 2.63 | 4.14 | 4.14 | 4.43 | 5.98 | 6.01 | 6.27 |
| Med Amine Hajji | 1.27 | 1.26 | 1.26 | 2.14 | 2.16 | 2.16 | 3.65 | 3.76 | 3.74 | 5.24 | 5.37 | 5.31 |
| Med Abbes Jbéli | 1.25 | 1.17 | 1.17 | 2.13 | 2.06 | 2.10 | 3.80 | 3.64 | 3.69 | 5.43 | 5.26 | 5.28 |
| Badis Daaji | 1.38 | 1.35 | 1.38 | 2.35 | 2.31 | 2.35 | 4.10 | 4.11 | 4.16 | 5.83 | 5.95 | 6.01 |
| Bechir Hosni | 1.41 | 1.47 | 1.41 | 2.40 | 2.56 | 2.41 | 4.24 | 4.44 | 4.26 | 6.04 | 6.40 | 6.10 |
| Akrem Aouini | 1.34 | 1.29 | 1.31 | 2.27 | 2.22 | 2.26 | 3.94 | 2.93 | 3.97 | 5.63 | 5.63 | 5.66 |
| Nabil Beji | 1.17 | 1.29 | 1.20 | 2.05 | 2.18 | 2.08 | 3.61 | 3.67 | 3.67 | 5.10 | 5.22 | 5.19 |
| Rayen Rafrafi | 1.28 | 1.32 | 1.12 | 2.14 | 2.16 | 1.99 | 3.68 | 3.71 | 3.56 | 5.31 | 5.24 | 5.15 |
| Med Amine Boudabbous | 1.24 | 1.25 | 1.28 | 2.18 | 2.23 | 2.24 | 3.99 | 4.11 | 4.01 | 5.59 | 5.91 | 5.80 |
| Saif Rourou | 1.28 | 1.20 | 1.26 | 2.19 | 2.06 | 2.18 | 3.79 | 3.71 | 3.77 | 5.34 | 5.31 | 5.35 |
| Med Malek Teji | 1.48 | 1.66 | 1.61 | 2.63 | 2.81 | 2.75 | 4.75 | 4.94 | 4.91 | 6.89 | 7.18 | 7.20 |
| Med Iheb Marsaoui | 1.47 | 1.43 | 1.38 | 2.55 | 2.45 | 2.36 | 4.52 | 4.27 | 4.25 | 6.53 | 6.17 | 6.24 |
| Ala Weraghni | 1.55 | 1.51 | 1.30 | 2.58 | 5.57 | 2.29 | 4.45 | 4.49 | 4.06 | 6.33 | 6.43 | 5.96 |
| Hassene Makni | 4.45 | 1.43 | 1.40 | 2.42 | 2.41 | 2.35 | 4.25 | 4.19 | 4.16 | 6.00 | 5.99 | 5.96 |
| Med Amine Aloui | 1.41 | 1.43 | 1.51 | 2.40 | 2.41 | 2.50 | 4.26 | 4.19 | 4.33 | 6.03 | 6.38 | 5.92 |
